# Incremental Mutual Information: A New Method for Characterizing the Strength and Dynamics of Connections in Neuronal Circuits

**DOI:** 10.1371/journal.pcbi.1001035

**Published:** 2010-12-09

**Authors:** Abhinav Singh, Nicholas A. Lesica

**Affiliations:** Ear Institute, University College London, London, United Kingdom

## Abstract

Understanding the computations performed by neuronal circuits requires characterizing the strength and dynamics of the connections between individual neurons. This characterization is typically achieved by measuring the correlation in the activity of two neurons. We have developed a new measure for studying connectivity in neuronal circuits based on information theory, the incremental mutual information (IMI). By conditioning out the temporal dependencies in the responses of individual neurons before measuring the dependency between them, IMI improves on standard correlation-based measures in several important ways: 1) it has the potential to disambiguate statistical dependencies that reflect the connection between neurons from those caused by other sources (e.g. shared inputs or intrinsic cellular or network mechanisms) provided that the dependencies have appropriate timescales, 2) for the study of early sensory systems, it does not require responses to repeated trials of identical stimulation, and 3) it does not assume that the connection between neurons is linear. We describe the theory and implementation of IMI in detail and demonstrate its utility on experimental recordings from the primate visual system.

## Author Summary

The root of our brain's computational power lies in its trillions of connections. With our increasing ability to study these connections experimentally comes the need for analytical tools that can be used to develop meaningful quantitative characterizations. In this manuscript, we present a new such tool, incremental mutual information (IMI), that enables the characterization of the strength and dynamics of the connection between a pair of neurons based on the statistical dependencies in their spiking activity. IMI is an important step forward from existing approaches, as it has the potential to disambiguate dependencies due to the connection between two neurons from those due to other sources, such as shared external inputs, provided that the dependencies have appropriate timescales. We demonstrate the utility of IMI through the analysis of simulated neuronal activity as well as activity recorded in the primate visual system.

## Introduction

To understand the function of neuronal circuits and systems, it is essential to characterize the connections between individual neurons. The major connections between and within many brain areas have been mapped through anatomical studies, but these maps specify only the existence of connections, not their strength or dynamics (temporal properties). Measuring the strength and dynamics of the connection between two neurons requires physiological experiments in which the activity of both neurons is measured. The most direct of these experiments involves intracellular recordings, which allow the connection between the two neurons to be directly investigated. However, intracellular recordings are difficult to perform *in vivo* and impossible to obtain from more than a few cells at a time. Instead, most physiological studies of connectivity rely on extracellular recordings from multi-electrode arrays (or, more recently, imaging of calcium activity). In these experiments, it is not usually possible to explicitly verify anatomical connectivity, nor to directly characterize the connections. Instead, the strength and dynamics of ‘functional’ connectivity must be inferred through statistical methods.

The traditional method for characterizing the strength and dynamics of the connection between two neurons is the cross correlation function (*C_XY_*), which measures the linear correlation between two signals over a range of specified delays [1]. While *C_XY_* and its variants have been used successfully in a number of studies (see, for example, Usrey and Reid [2] for a review of many such studies in the visual system), it has limitations that must be considered when studying the connection between neurons [3]–[5]. The limitations of *C_XY_* arise from the fact that it is a measure of the total (linear) dependency between two signals and, thus, implicitly assumes that all dependencies between them are due to their connection. In the case of neurons, there are in fact many potential sources of dependency – shared external stimuli, intrinsic cellular and network properties, etc. – and *C_XY_* cannot disambiguate these dependencies from those due to the actual connection. Several modified versions of *C_XY_* have been proposed to address these drawbacks. For example, if neuronal activity in response to repeated trials of the same external stimulus is available for analysis, as is often the case in early sensory systems, the ‘shift-predictor’ can be used to remove some of the correlations due to the stimulus [1]. Further modifications to *C_XY_* have also been proposed to remove the correlations due to stimulus-driven covariations in activity [6] and background activity [7]. While these modified approaches have certainly improved upon the standard *C_XY_*, the confound of dependencies due to the connection and those arising from other sources remains a general problem.

In addition to correlation-based methods, there are several other approaches to characterizing the dependency between two signals that can be used to study the connection between two neurons. These methods can be generally divided into two classes: model-based and model-free. The most common model-based approach to characterizing dependency is Granger causality (GC) [8]. With GC, one signal is predicted in two different ways: 1) using an autoregressive model based on its own past and 2) using a multivariate autoregressive model based on its own past and the past of the second signal. The strength of the dependency is given by the difference in the predictive power of the two models and the dynamics of the dependency are reflected in the regression parameters that correspond to the influence of the second signal. The power of model-based approaches such as GC is dependent on the validity of the underlying model; if the dependency between the two signals is approximately linear, then the characterization provided by GC will be accurate, but in situations where the properties of the dependency are complex or unknown, as is often the case with neurons, a model-free approach may be more appropriate. The most common model-free approach to characterizing dependency is transfer entropy (TE), the information-theoretic analog of GC [9]. TE measures the reduction in the entropy of one signal that is achieved by conditioning on its own past and the past of the second signal relative to the reduction in entropy achieved by conditioning on its own past alone. TE is a powerful tool for measuring the overall strength of a dependency, but is not suitable for characterizing its dynamics.

In this paper, we detail a new model-free approach for characterizing both the strength and dynamics of a dependency by ‘conditioning out’ the temporal correlations in both signals before assessing the strength of the dependency at different delays. This approach can overcome some of the confounds that are common in studies of neuronal connectivity [10]–[12], as it has the potential to disambiguate statistical dependencies that reflect the connection between neurons from those caused by other sources (e.g. shared inputs or intrinsic cellular or network mechanisms) provided that the dependencies have appropriate timescales. In the following sections, we outline the theory behind our measure, which we call incremental mutual information, illustrate its usage on simulated neuronal activity and experimental recordings from the primate visual system, and consider its relationship to other common measures of dependence.

Matlab code for measuring incremental mutual information is available for download at http://www.ucl.ac.uk/ear/research/lesicalab

## Methods

### Correlation

In order to characterize the strength and dynamics of the connection between two signals, it is necessary to quantify how much one signal at one point in time influences the other signal at nearby points in time. Most measures of dependence between two signals *X* and *Y* seek to quantify the difference between the joint distribution *p*(*X,Y*) and the product of the marginal distributions *p*(*X*) *p*(*Y*). For example, the cross correlation function measures the difference between the mean of the joint distribution and the product of the means of the marginal distributions (the covariance), normalized by the product of the standard deviations for a given delay *δ*:
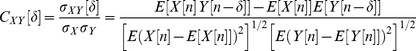
(1)where *C_XY_*[*δ*] is the correlation coefficient between *X*[n] and *Y*[n], which are assumed to be discretized signals, at integer delay *δ*.

### Partial correlation

**Figure 1:**
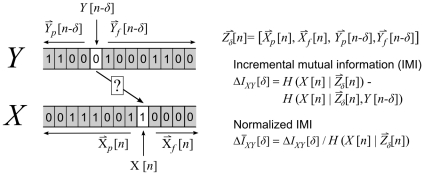
The quantities involved in computing incremental mutual information. The incremental mutual information (IMI) between two signals *X* and *Y* is computed by first computing the entropy of *X*[*n*] after conditioning on 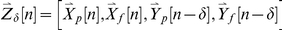, a vector comprised of the past and future of both signals relative to a delay *δ*. This entropy is then compared the entropy of *X*[*n*] after further conditioning on *Y*[*n−δ*]. The reduction in entropy due to this further conditioning is the incremental mutual information.

As described in the Introduction, *C_XY_* has limitations that are important to consider when studying neuronal connectivity. Most importantly, *C_XY_*, as with all dependency measures that operate only on the joint distribution *p*(*X,Y*) and the marginal distributions *p*(*X*) and *p*(*Y*), cannot differentiate between the dynamics of the connection between the neurons and the temporal correlations in their activity that are due to other sources. It is possible to overcome this limitation by conditioning out the temporal correlations in each signal before measuring the dependency between them, i.e. rather than operate on *p*(*X,Y*), *p*(*X*), and *p*(*Y*), operate on *p*(*X,Y*|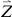), *p*(*X*|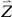), and *p*(*Y*|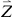), where 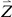 is a vector containing the past and future of *X*[n] and *Y*[n] relative to the delay of interest
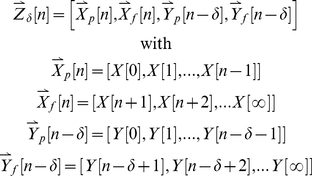
(2)as shown in the schematic diagram in figure 1.

The analog of *C_XY_* for conditional distributions is the partial cross correlation:
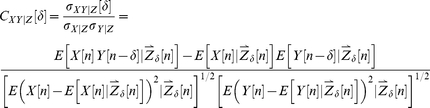
(3)While *C_XY|Z_* overcomes the major limitation of *C_XY_*, it is still a linear measure and may not accurately characterize nonlinear dependencies.

### Incremental mutual information

The idea of partial correlation can be generalized for the study of any dependency by formulating the information-theoretic analog of *C_XY|Z_* as a partial mutual information [13]: First, the entropy of *X* is measured after conditioning on its own past and future, as well as the past and future activity of *Y* relative to the delay of interest. Then, the strength of the influence of *Y* on *X* at the delay of interest can be measured as the additional reduction in entropy that occurs after observing *Y* at that delay:
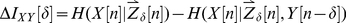
(4)Because this quantity, which we call the incremental mutual information (IMI), reduces the uncertainty of *X* as much as possible before measuring the influence of *Y* at each delay, it has the potential to provide an accurate description of both the strength and dynamics of their dependency. In this form, Δ*I_XY_* is similar to a partial covariance in that its value is dependent on the properties of the individual signals (e.g. the total entropy of *X*, the strength of the temporal correlations in *X*, etc.). In some cases, it may be preferable to use a normalized measure that is more similar to a partial correlation coefficient, i.e. a measure that expresses the incremental mutual information as a fraction of its maximum possible value:
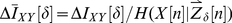
(5)To determine whether IMI is appropriate for use in any particular context, it is important to consider the relative timescales of the dependency between the signals and the other dependencies to be conditioned out. At any particular delay, the effects of dependencies with durations that are long relative to the time bins used for discretization will be predictable from the past and future values of the signals, so their contribution to the IMI will be small, i.e. dependencies with a slow timescale will make a relatively large contribution to initial reduction in the entropy of *X* based on past and future values of *X* and *Y*, 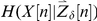, but not to the additional reduction in the entropy of *X* based on the present value of *Y*, 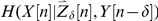. Conversely, the effects of dependencies that have a duration that is similar to the time bins used for discretization will not be predictable from the past and future values of the signals, so their contribution to the IMI will be large, i.e. dependencies with a fast timescale will make a small contribution to the initial reduction in entropy 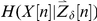, but will make a large contribution to the additional reduction in entropy 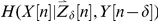. Thus, IMI will be most useful when the duration of the dependency between the signals is similar to the size of the time bins used for discretization and the durations of the other dependencies to be conditioned out are longer. Fortunately, this is often the case for neurons in sensory systems, as will be illustrated in the examples in the Results.

### Implementation

As with any measure based on entropies, the calculation of IMI requires careful consideration. Because IMI is a model-free approach, the number of samples required to produce a result of a given precision are likely to significantly exceed those of model-based approaches. The bias and variability of the entropy estimates that underlie the computation of IMI can vary substantially depending on the size of the data sample, the number of possible values that a signal can take on, and the signal dimensionality. Fortunately, neuronal activity typically has only a few possible values (e.g. the number of spikes in each time bin). However, the terms 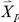, 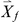, 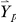, and 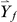 representing the past and future of the signals are vectors. In practice, these vectors must be limited to some finite length, which we term *ω*, and this length will determine their dimensionality:
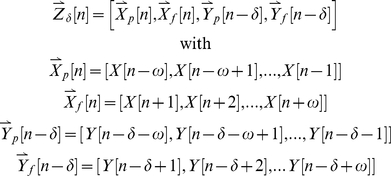
(6)Thus, the calculation of IMI requires a tradeoff: increasing the value of *ω* allows the entropy of the first signal to be reduced as much as possible before measuring the influence of the second signal, but also increases the chances that the entropy estimates may be biased or highly variable. There are a number of bias correction techniques available that may be useful in mitigating problems related to sample size [14]. For the examples below, we corrected the entropy estimates using ‘quadratic extrapolation’ bias correction via the information toolbox software available at http://www.ibtb.org [15]. Also, for all of the examples below, time is discretized into sufficiently small bins such that each bin contains no more than one spike, limiting the possible values of *X* and *Y* to 0 and 1.

### Statistical inference

Because the bias and variability of entropy estimates are dependent on sample size, it is critical to establish the validity and precision of any calculation of IMI using statistical methods. In the experimental examples presented below, we use two different bootstrap procedures with random sampling to establish 95% confidence intervals and to determine whether the observed values are significantly different from zero. To establish 95% confidence intervals, we calculated IMI from 100 random samples of the same size drawn with replacement from the original sample. To preserve the temporal dependencies in the data, sampling was performed after the vectors 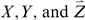 were formed and the three vectors were sampled together. Confidence intervals were defined as the mean ± 2 standard deviations of the values calculated from the random samples. To establish the significance of the observed values, the same procedure was followed, but *Y* was sampled separately from 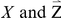. This sampling preserved the dependencies between 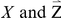, but removed the dependencies between *X* and *Y* (and, thus, in theory, removed any IMI between them). The observed values were considered significantly different from zero if they were greater than 2 standard deviations above the mean of the values calculated from the random samples.

## Results

### Simulated example 1: Differentiating input correlations and connection dynamics

**Figure 2:**
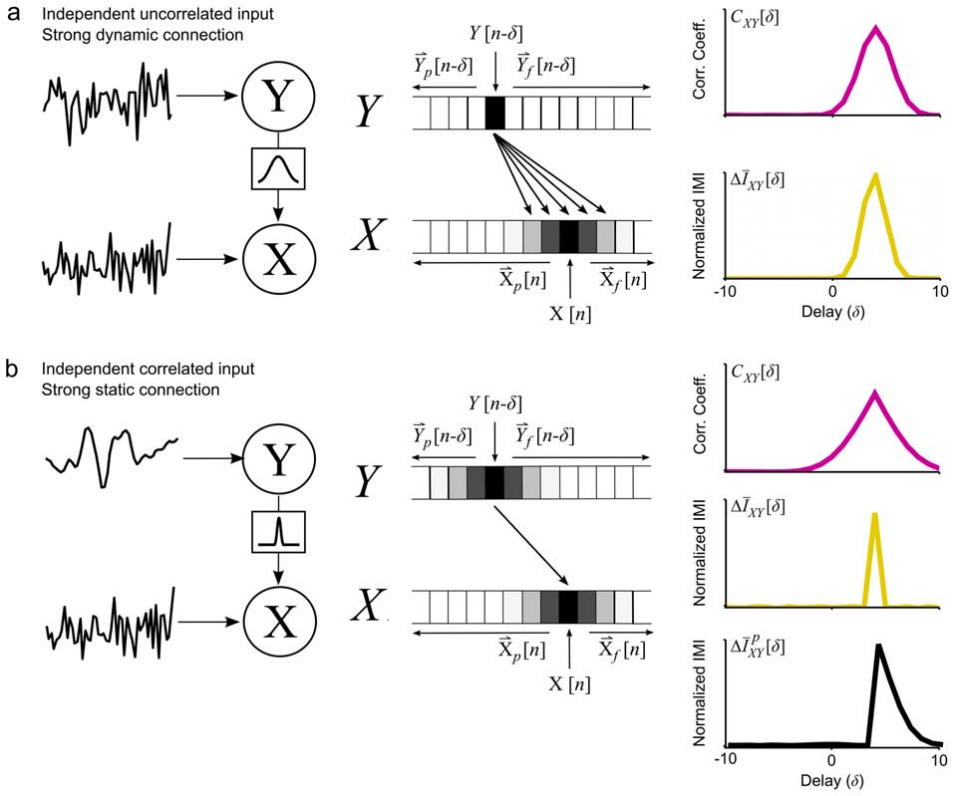
Incremental mutual information disambiguates temporal correlations and connection dynamics. a) A schematic diagram showing two neurons *X* and *Y*. The two neurons are driven by independent uncorrelated noise sources and *Y* drives *X* through a strong dynamic connection. The cross correlation function *C_XΥ_* and normalized IMI 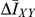 computed from the simulated activity of the two neurons at a range of delays are shown. b) A second pair of neurons *X* and *Y*. The two neurons are driven by independent noise sources. The source driving *Y* has temporal correlations while the source driving *X* is uncorrelated. *Y* drives *X* through a strong static connection with a delay of 4 samples. The cross correlation function *C_XΥ_*, the normalized IMI 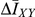, and the normalized IMI with only past activity conditioned out 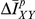 computed from the simulated activity of the two neurons at a range of delays are shown. IMI was computed with *ω* = 2 for 2^20^ samples.

IMI is designed to give accurate measures of the strength and dynamics of the connections between neurons even in cases when the correlation may not, i.e. when the activities of individual neurons contain temporal correlations unrelated to the connection between them. In these cases, the cross correlation function can be ambiguous – its shape can reflect either the true dynamics of the connection, temporal correlations in the activities of the individual neurons, or a combination of both. A simple example of this ambiguity is illustrated in figure 2a.

We first simulated a pair of neurons *X* and *Y* with independent, uncorrelated inputs and a dynamic connection, i.e. a spike from neuron *Y* caused a prolonged increase in the spiking probability of neuron *X*. We simulated the activity of neuron *Y* as a dichotomized Gaussian noise and the activity of neuron *X* as the dichotomized sum of a Gaussian noise and the filtered activity of *Y*:
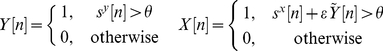
(7)where 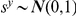 and 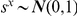 are uncorrelated, *ε* = 0.5 is a scaling factor determining the overall strength of the connection, *θ* = 1 is the spiking threshold, and the input from *Y* to *X*, 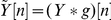, is the convolution of the activity of *Y* with a Gaussian filter *g*[*n*] with a peak delay of 4 samples and a half width of 3 samples (note that 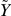, the filtered version of *Y*, is unobserved). From the simulated activity of this pair of neurons (with a sample size of 2^20^), we estimated the cross correlation function *C_XY_* and normalized incremental mutual information 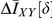 (with *ω* = 2) at delays ranging from *δ* = −10 to 10 samples. Both *C_XY_* and 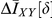 for this pair were broad, reflecting the dynamics of the connection.

We next simulated another pair of neurons that was similar to the first one, except that *Υ* received input with temporal correlations and the connection between *Υ* and *X* was static with a delay of 4 samples:
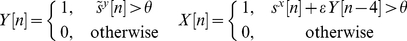
(8)where 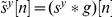 is the convolution of *s^y^* with a Gaussian filter *g*[*n*] with a peak at zero delay and a half width of 3 samples. While *C_XY_* for this pair was also broad because of the temporal correlations in the activity of *Y*, 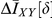 was sharp, reflecting the static connection. Thus, while IMI captures the differences in the connections between these two pairs of neurons, correlation conflates connection dynamics with temporal correlations in individual activities and yields ambiguous results.

This example can also be used to illustrate the necessity of conditioning out the both past and future activities of the neurons. A modified version of IMI can be formulated in which only the past activities of the two neurons are conditioned out:
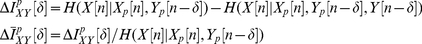
(9)In this formulation, the IMI is related to transfer entropy (see Discussion). As shown in figure 2b, 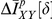 correctly conditions out the effects of the temporal correlations in the activity of *Y* for delays that are smaller than that of the actual connection, but not for delays that are larger than that of the actual connection. This reason for this asymmetry is as follows: Because of the temporal correlations in the activity of *Y*, its value will be similar for neighboring samples. When the delay of interest *δ* is smaller than the delay corresponding to the actual connection δ^*^, *Y*[*n*−δ^*^] is included in the vector of past activity and, since *Y*[*n*−*δ*] carries no information about *X* beyond that which is carried by *Y*[*n*−δ^*^], *Y*[*n*−*δ*] makes no contribution to the IMI. However, when *Y*[*n*−δ^*^] is not included in the vector of past activities, *Y*[*n*−*δ*], which is similar to *Y*[*n*−δ^*^] because of the temporal correlations in *Y*, will carry additional information about the activity of *X* and, thus, will contribute to the IMI.

### Simulated example 2: Unmasking a weak connection

**Figure 3:**
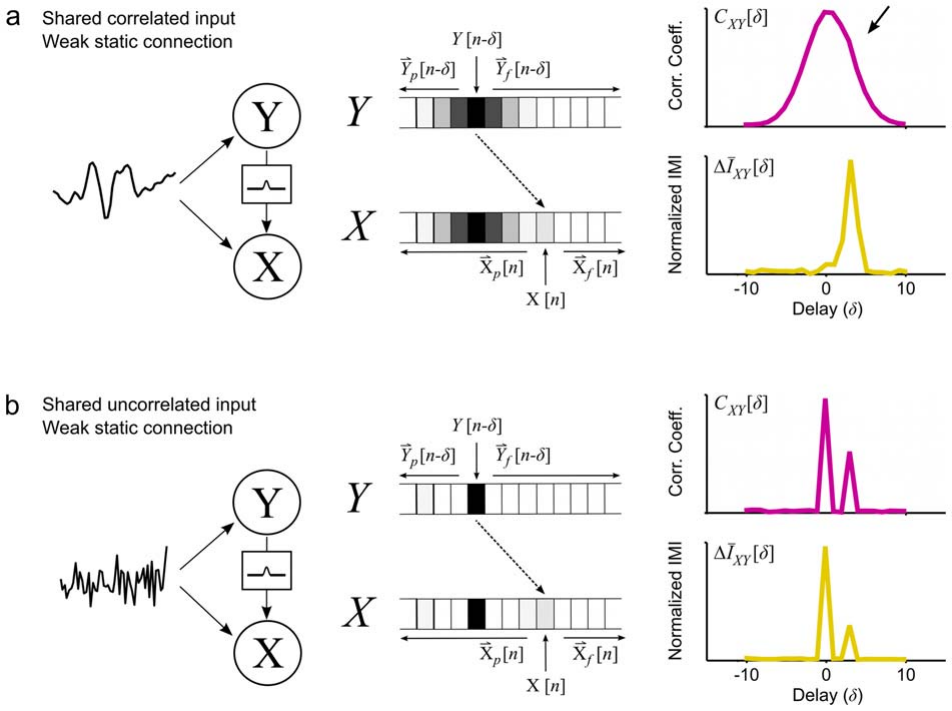
Incremental mutual information unmasks weak connections. a) A schematic diagram showing two neurons *X* and *Y*. The two neurons are driven by a shared correlated noise source and *Y* drives *X* through a weak static connection. The cross correlation function *C_XΥ_* and normalized IMI 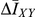 computed from the simulated activity of the two neurons at a range of delays are shown. b) Results for the same simulated neurons driven by a shared uncorrelated source, presented as in panel a. IMI was computed with *ω* = 2 for 2^20^ samples.

As a further consequence of the ambiguity in the cross correlation function illustrated in the example above, temporal correlations in individual activities may mask weak connections between neurons entirely. A simple example of this problem is shown in figure 3a. We simulated a pair of neurons that received a shared input with temporal correlations and had a weak static connection between them with a delay of 3 samples:
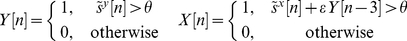
(10)where 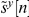 and 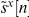 are the convolution of Gaussian noise with a Gaussian filter as described above with a correlation coefficient of 0.5 between them, and *ε* = 0.25 (other parameter values are as described above). *C_XY_* for this pair of neurons was broad, with no discernable increase at the delay corresponding to the connection (black arrow), while 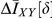 exhibits a sharp peak at the appropriate delay. Thus, by conditioning out dependencies due to shared input, IMI is able to reveal connections that may not be evident in the cross correlation function.

### Simulated example 3: Shared inputs that cannot be conditioned out

A slight modification of the previous example can be used to illustrate a situation where shared inputs cannot be conditioned out and contaminate the IMI. As described above, IMI will be most useful when the duration of the dependency between the signals is similar to the size of the time bins used for discretization and the durations of the other dependencies to be conditioned out are longer, as is the case in example 2. If the simulation in example 2 is modified so that the shared input is uncorrelated over time, the dependency resulting from the shared input can no longer be conditioned out, as the past and future activities of the neurons can no longer be used to infer the effects of the input at the delay of interest. As a result, 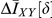 has two peaks, one with no delay reflecting the shared input, and another with a delay reflecting the actual connection, as shown in figure 3b. It should be noted that this type of contamination can potentially arise both from shared external sources such as sensory stimuli as well as from other unobserved neurons.

### Experimental example 1: Thalamic relay neurons and their retinal inputs

**Figure 4:**
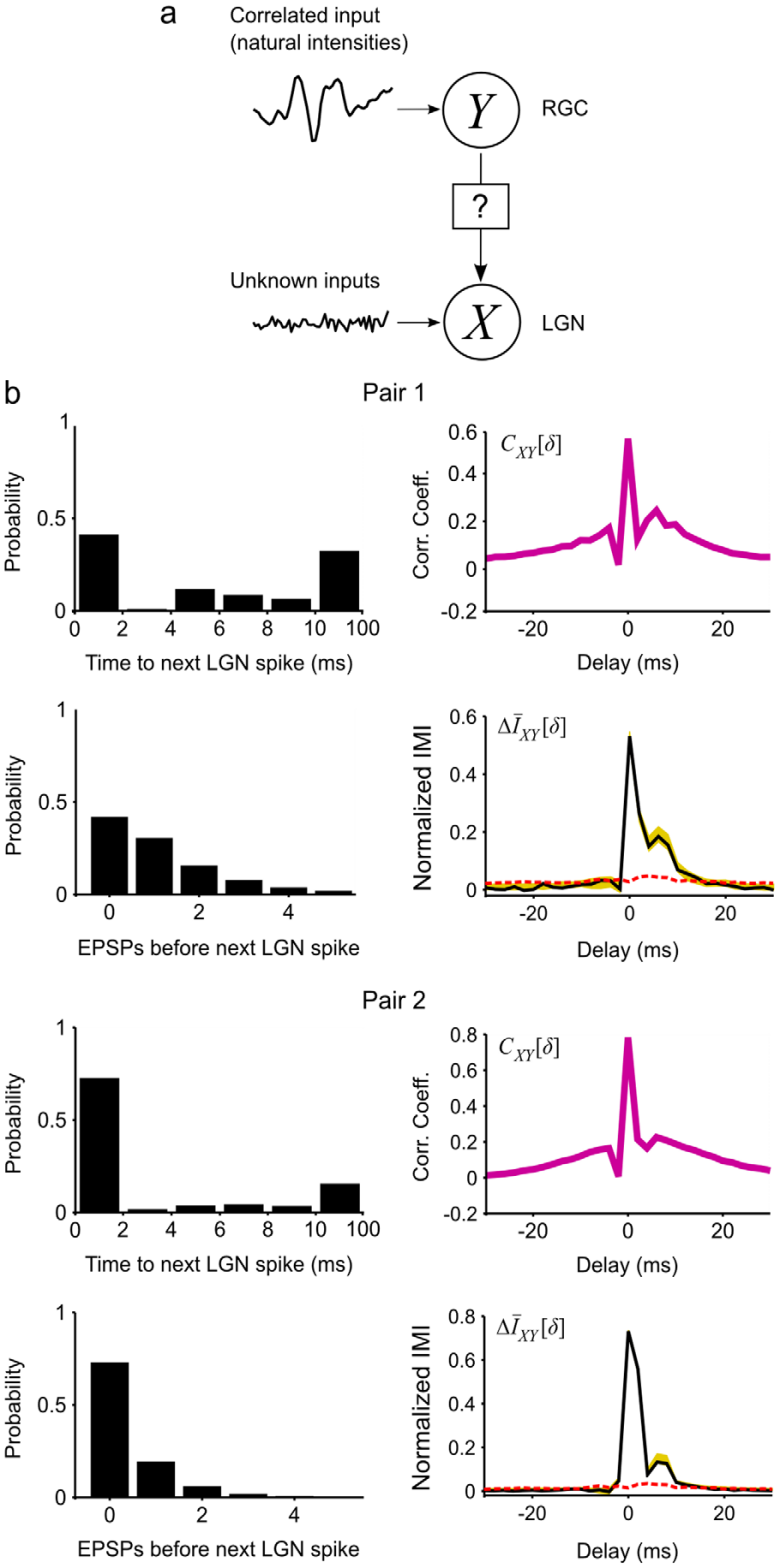
Incremental mutual information analysis of retinogeniculate pairs. a) A schematic diagram showing two neurons *X* and *Y*. *Y* is a retinal ganglion cell driven by a stimulus with temporal correlations that are typical of the natural environment. *X* is an LGN relay cell driven by *Y* and an unobserved noise source. b) Histograms showing the distribution of time delays between each retinal PSP and the next LGN spike and the number of additional retinal PSPs that preceded the next LGN spike, as well as the cross correlation function *C_XΥ_* and normalized IMI 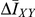 computed from the responses of two retinogeniculate pairs to a non-repeating stimulus at a range of delays. For the IMI, the black line indicates the actual estimate, the yellow band indicates 95% confidence intervals, and the red dashed line indicates the significance level. Confidence intervals and significance levels were generated via bootstrap procedures with random sampling as described in the Methods. Spike times were binned with a resolution of 2 ms and IMI was computed with *ω* = 4 for approximately 2^18^ samples.

To test the utility of IMI on experimental data, we analyzed the activity of two pairs of thalamic relay neurons and their retinal ganglion cell (RGC) inputs recorded in the lateral geniculate nucleus (LGN) of an anesthetized monkey as shown in figure 4a. The details of the experimental procedures can be found in Carandini et al. [16]. During the recordings, visual stimulation was presented via an LED that illuminated the receptive field center with an intensity that varied naturally (i.e. with temporal correlations typical of the natural environment). In this example, the stimulus was approximately 12 min in duration and did not repeat.

The histograms in figure 4b show the basic relationship between the activity of the retinal and thalamic neurons in each pair. For the first pair, less than half of the RGC postsynaptic potentials (PSPs) evoked immediate LGN spikes, while the connection between the second pair was stronger, with nearly 75% of PSPs evoking immediate spikes. We calculated the cross correlation function and incremental mutual information for these pairs after binarizing the spike trains in 2 ms time bins. *C_XY_* for these pairs has a complex shape with 3 components: a broad positive peak with a half width of approximately 20 ms reflecting the temporal correlations in the visual stimulus, two sharp negative peaks reflecting refractory effects, and a sharp positive peak reflecting the actual connection between the cells. In contrast, 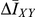 for these pairs had one main peak reflecting the connection between the neurons - the effects of statistical dependencies arising from the stimulus correlations have been completely removed and the refractory effects have been largely conditioned out. For the first pair, 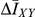 had a relatively long tail, reflecting temporal summation of RGC PSPs that failed to evoke an immediate LGN spike. For the second pair, 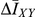 was sharper, reflecting the stronger connection between the cells.

### Relation between incremental mutual information and signal and noise correlations

In early sensory systems, experiments are often designed such that the activity in response to repeated trials of an identical stimulus are observed so that the correlation between neurons can be separated into two distinct parts known as *signal correlation* and *noise correlation*. The signal correlation, which reflects both correlation in the stimulus itself and similarities in neurons' preferred stimulus features, will capture the correlation in the fraction of the response that is repeatable from trial to trial, i.e. the correlation that remains after the trial order has been randomized:
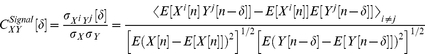
(11)where *X^i^*[*n*] is the response of neuron X on trial *i* and 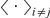 indicates the expectation over all possible combinations of trials *i* and *j* in which their values are not equal. In studies of neuronal connectivity, 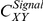 is often referred to as the ‘shift-predictor’.

The noise correlation, which results from network and intrinsic cellular mechanisms, will capture the remaining correlation in the fraction of the response that is variable from trial to trial
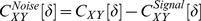
(12)and, thus, captures the dependencies between the neurons that are not locked to the external stimulus. However, while 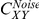 may provide a better measure of the strength and dynamics of the connection between two neurons than *C_XY_*, it still confounds connection dynamics and temporal correlations that are independent of the stimulus, e.g. refractory effects or coupled oscillations.

For comparison with 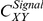 and 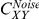, the signal and noise IMI between *X* and *Y* can be formulated in an analogous fashion. The signal IMI is the reduction in the entropy of the response of *X* on trial *i* that results from observing the response of *Y* on trial *j* at the delay of interest, beyond that which results from observing the past and future responses of both neurons on trial *i*:
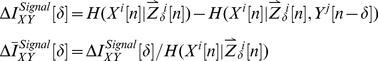
(13)where 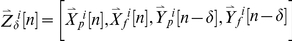. The noise IMI is the difference between the total IMI and the signal IMI, i.e. the reduction in the entropy of the response of *X* on trial *i* that results from observing the response of *Y* on trial *i* at the delay of interest and the past and future responses of both neurons on trial *i*, beyond that which results from observing the response of *Y* at the delay of interest on trial *j* and the past and future responses of both neurons on trial *i*:
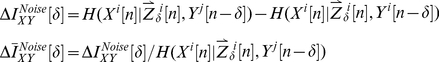
(14)


### Experimental example 2: Thalamic relay neurons and their retinal inputs revisited

**Figure 5:**
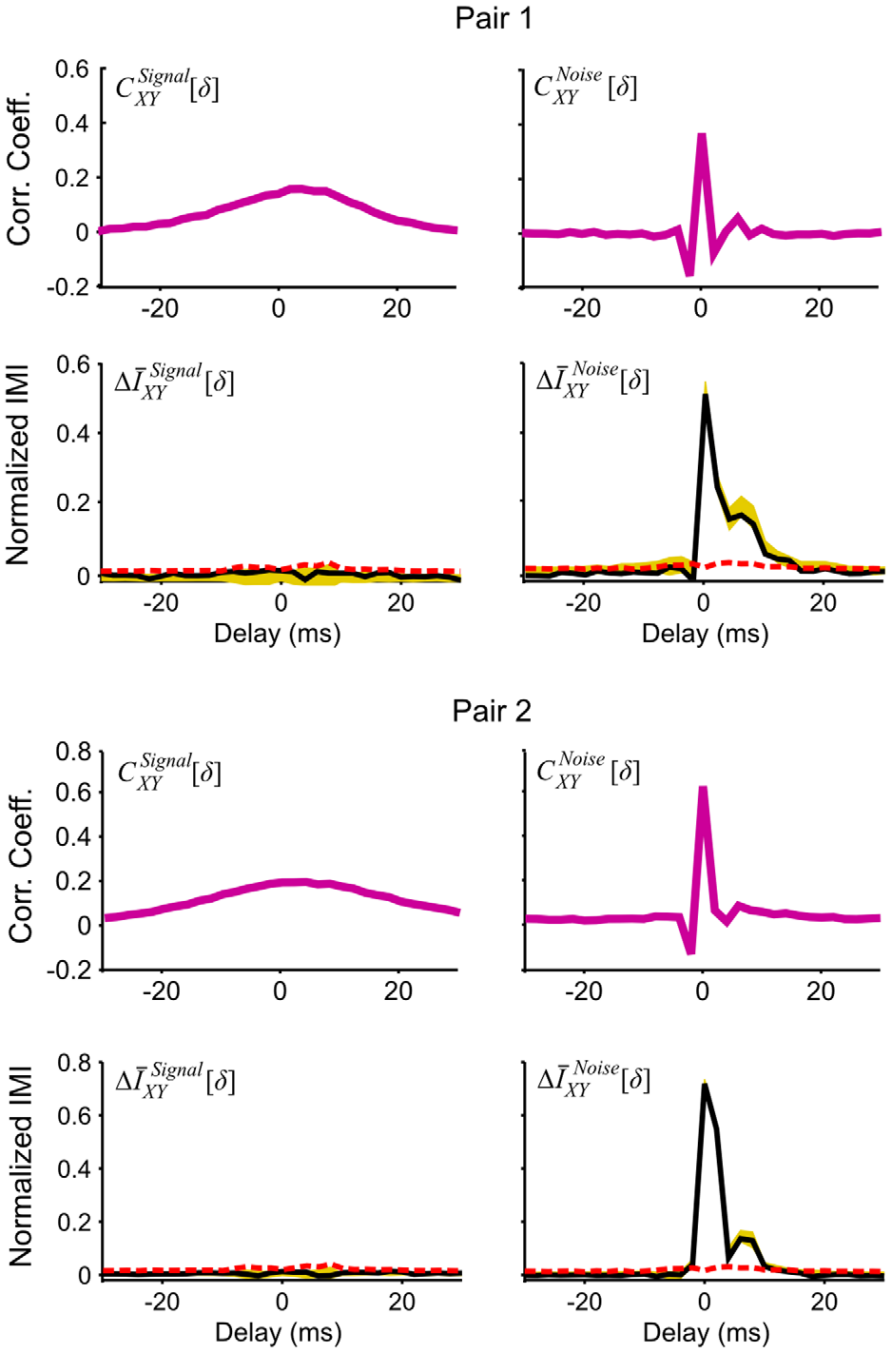
Signal and noise incremental mutual information. The signal and noise cross correlation functions 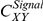 and 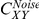 and the normalized signal and noise IMI 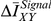 and 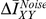 computed from the responses of the same two retinogeniculate pairs as in figure 4b to repeated trials of an identical stimulus, presented as in figure 4b. Spike times were binned with a resolution of 2 ms and IMI was computed with *ω* = 4 for approximately 2^18^ samples.

We estimated the signal and noise correlations and signal and noise IMI for the same two retinogeniculate pairs that were analyzed in experimental example 1 using a different set of responses to 140 repeated trials of identical stimulation in which each trial was 5 seconds in duration. As shown in figure 5, 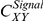 for both pairs was broad, reflecting the temporal correlations in the visual stimulus. In contrast, 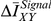 was nearly zero at all delays – because the temporal correlations in the visual stimulus were slow relative to the bin size used for discretization, there was little information about stimulus-induced dependencies to be gained by observing the RGC activity at any particular delay on a different trial when RGC and LGN activity at surrounding delays on the current trial were already known. While the effects of the stimulus correlations were removed from 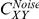 for both pairs, these functions still had a complex shape, with two negative peaks reflecting refractory effects, and one positive peak reflecting the actual connection between the neurons. Thus, while shuffling removed some of the confounding correlations in *C_XY_*, others still remained, while in 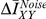, which has one main peak reflecting the connection between the neurons, most of the confounding dependencies have been conditioned out.

This example illustrates an important property of IMI. 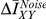 as shown in figure 5 is nearly identical to 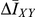 for the same two pairs shown in figure 4. Thus, unlike the cross correlation function, IMI does not require multiple trials in order to differentiate the temporal correlations in the responses of individual neurons from the dynamics of the connection between them.

## Discussion

We have presented IMI as a new approach to characterizing the strength and dynamics of the connection between neurons. By conditioning out the temporal dependencies in the responses of individual neurons before assessing the connection between them, IMI improves on correlation-based measures in several important ways: 1) IMI has the potential to disambiguate connection dynamics from other temporal dependencies due to shared inputs or intrinsic cellular or network mechanisms provided that the dependencies have appropriate timescales, 2) for the study of sensory systems, IMI does not require responses to repeated trials of identical stimuli, and 3) IMI does not assume that the connection between neurons is linear. Through example applications of IMI to simulated and experimentally recorded neuronal activity, we have demonstrated that IMI has the potential to be both a powerful and practical tool for analyzing the functional connectivity in neuronal circuits.

### Limitations

The major determinant of the ability of IMI to differentiate connection dynamics from other dependencies is the relative timescale of the other dependencies. If the other dependencies have a long duration relative to the time bins used for discretization, then their effects can be conditioned out through observation of past and future neuronal activity, as demonstrated in the experimental examples presented above. If the other dependencies have a duration that is similar to the bin size, then their effects cannot be conditioned out without explicit observation of their source.

As formulated here, IMI is designed to analyze the connection between a pair of neurons. However, in many brain areas, each neuron receives input from a large population, and correlations between these other inputs and the input under study could contaminate the IMI. If the other inputs are unobserved, it will be difficult to account for their effects with a model-free approach, though recent work with model-based approaches has demonstrated some success [17]–[20]. If the other inputs are observed (which is becoming increasingly common with recent advances in recording and imaging technology that allow for simultaneous recording of the activity complete or nearly complete local populations of neurons), there is no reason that, in principle, IMI cannot be extended to condition out dependencies due to the activity of the other neurons. However, adding the activity of additional neurons to the conditioning vector 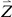 will increase its dimensionality, and, thus, the bias and variability of the entropy estimates that underlie the computation of IMI. While this may not be a problem for a small number of neurons, it is certain to be a problem for large populations. Thus, for large populations, it may be more appropriate to use a model-based approach such as Granger causality within a generalized linear model framework [21].

### Relation between incremental mutual information and transfer entropy

Of the existing approaches to characterizing dependencies between signals, IMI is most similar to transfer entropy [9]. TE measures the dependency between two signals as the difference in the entropy of one signal after conditioning on its own past and conditioning on its own past and the past of the other signal, or, in the terminology used to define IMI, 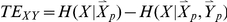. From this definition, it is clear that TE and IMI are designed for different purposes: TE measures the overall causal strength of the dependency between two signals by first conditioning out the past of one signal and then measuring how much can be learned about the present value of that signal based on the past of the second signal, while IMI measures the strength and dynamics of the dependency between two signals by first conditioning out past and future of both signals and then measuring how much can be learned about the present value of one signal from the present value of the other relative to some delay. The key difference between TE and IMI, as illustrated in the simulated example presented above, is that, even if computed at a range of delays, TE is not suitable to assess the dynamics of a dependency because it considers only past activity and, as a result, conditions out temporal correlations appropriately for delays that are shorter than that of the actual dependency, but not for delays that are longer than that of the actual dependency.

### Relation between IMI and generalized linear models

The most effective model-based approach for studying the functional connectivity in a neuronal circuit is the generalized linear model (GLM) [22]–[24]. The GLM attempts to predict a neuron's activity based not only on its own activity and the activity of other neurons, but also on external inputs. Because all of the filters in the model are fit simultaneously, the influence of the external inputs on the activity of each neuron, as well as those of its own past activity, are separated from the influence of other neurons. The power of the GLM lies in the fact that once the filters have been estimated, the model can be used to predict the activity of the entire group of neurons to any external input, but this power comes at the expense of assuming a particular parametric structure. Relative to IMI, which makes no assumptions about the connections between neurons, the drawback of the GLM is that the interactions between neurons are assumed to be of a particular nature (usually additive). However, this assumption also allows the GLM to be readily applied to large populations.
